# Cost-Normalized Circular Economy Indicator and Its Application to Post-Consumer Plastic Packaging Waste

**DOI:** 10.3390/polym13203456

**Published:** 2021-10-09

**Authors:** Rafay Tashkeel, Gobinath P. Rajarathnam, Wallis Wan, Behdad Soltani, Ali Abbas

**Affiliations:** 1 School of Chemical and Biomolecular Engineering, The University of Sydney, Sydney, NSW 2006, Australia; rafaytashkeel@gmail.com (R.T.); wwan6910@uni.sydney.edu.au (W.W.); behdad.soltani@sydney.edu.au (B.S.); 2 Mercularis Pty Ltd., Sydney, NSW 2145, Australia

**Keywords:** circular economy, circo-economics, material circularity indicator, plastic waste, packaging

## Abstract

This work presents an adaptation of the material circularity indicator (MCI) that incorporates economic consideration. The Ellen MacArthur Foundation (EMF) has developed the MCI to characterize the sustainability, viz., the “circularity”, of a product by utilizing life cycle assessment data of a product range rather than a single product unit. Our new “circo-economic” indicator (MCIE), combines product MCI in relation to total product mass, with a cost-normalization against estimated plastic recycling costs, for both separately collected and municipal solid waste. This is applied to assess Dutch post-consumer plastic packaging waste comprising polyethylene (PE), polypropylene (PP), polyethylene terephthalate (PET), film, and mixed plastic products. Results show that MCIE of separate plastic collection (0.81) exceeds municipal solid waste (0.73) for most plastics, thus suggesting that under cost normalization, there is greater conformity of separately collected washed and milled goods to the circular economy. Cost sensitivity analyses show that improvements in plastic sorting technology and policy incentives that enable the production of MSW washed and milled goods at levels comparable to their separately collected counterparts may significantly improve their MCI. We highlight data policy changes and industry collaboration as key to enhanced circularity—emphasized by the restrictive nature of current Dutch policy regarding the release of plastic production, recycling, and costing data, with a general industry reluctance against market integration of weight-benchmarked recycled plastics.

## 1. Introduction

### 1.1. Plastics and Material Circularity

Current industrial metabolic patterns provide credence to the notion that the scale of current material production in the linear economy is unsustainable and that the circular economy is a key step in the establishment of sustainable industrial practices [1,2,3]. At its core, the circular economy concept refers to the various business practices, activities, and strategies used by organizations to minimize the demand for raw material inputs through the reduction, reuse, and recycling of materials back into production processes [2]. 

Despite an increasing interest in the circular economy among the scientific community over the last decade, few studies have focused on developing and assessing methodologies used to evaluate the “circularity” of product ranges, supply chain processes, and organizational-based services [4]. Namely, whilst several works have assessed the application of material circularity throughout various case studies, such analysis of products and organizations is still in its relative infancy, with a recent study demonstrating that only 10 out of 155 reviewed studies provided any focused critique of the indicators used for the assessment of circular-economy-based strategies [1,4,5]. This lack of quantification of the impacts of the circular economy is a prevalent issue despite current research that explicitly emphasizes the need for effective practical indicators to better describe “circularity” and thus facilitate the transition of organizations from linear to circular economy models [4,5]. 

**Figure 1:**
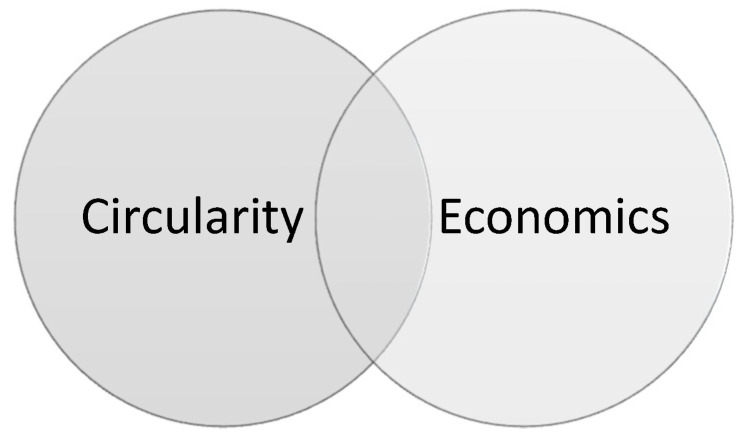
The concept of “circo-economics” is the common space merging material circularity with economics.

Considering the circular plastic economy, and specific example waste streams such as post-consumer PPW, international entities such as the European Commission’s circular economy package program have prioritized the reduction of plastic waste to landfill via the promotion of the recycling and optimization of post-consumer PPW in alignment with governmental entities, such as the European Parliament placing emphasis on plastic waste reduction efforts since 1994, in line with increased lack of space and consumer awareness [6,7]. Despite these efforts, previous research has identified that in nations such as the Netherlands, which are normally renowned for their innovation and implementation of national and European waste management strategies, the realistic rates of PPW recycling and recomposition lay at only 24% and 27%, respectively, with the remaining majority of PPW being incinerated for energy and heat production [7]. This was done despite recent research suggesting that the rerouting of plastic packaging waste to recycling facilities claimed to be a better environmental alternative than PPW incineration and landfilling [7,8]. Furthermore, despite researchers encouraging the use of MCIs for the evaluation of waste-derived products and packaging, there currently remains an overt emphasis in the current literature on the assessment of conventional aspects that are linked to the circular economy, such as CO_2_ emissions and waste production, while research into the circular economy performance indicators themselves is lacking, particularly the economics of material circularity—what we label as “circo-economics” (Figure 1). This incorporation of economics into the MCI is explored in this present work.

### 1.2. Challenges to the Measurement of Material Circularity

#### 1.2.1. Formulation of Integrated Sustainable Material Management (SMM) Options

For the implementation of sustainable materials management, comprehensive material life cycle data are crucial to adequately model the complex material life cycle [9]. This is relatively difficult to obtain due to the skills, knowledge, communication, and time required to collect material life cycle information and integrate the knowledge into the formation of a suitable visualization such as a Sankey diagram [9]. Systematic material flow analysis (MFA) techniques are the tool used to model material life cycle flows with multiple studies, resulting in prioritizing resource management opportunities ranging from the local to global level [9,10]. Additional complexity arises from reaching a conclusive Sankey model with the material flow analysis data due to the range of skills in systems science, material flow inventory curation, data analysis, and modelling being required to produce a validated flow of a material in the economy [9]. This, in turn, culminates in increased difficulties for sustainable materials management and decision making. The lack of comprehensive data for MFA therefore presents the core challenge in quantifying material circularity. 

#### 1.2.2. Definition of “Circularity”

Prior examination of the scientific literature on the circular economy demonstrates that a lack of specific definitions and criteria currently exists for the analysis and assessment of benefits, and measures for improvement and optimization of the circular economy [1]. Researchers such as Haas et al. [1] often utilize simplified definitions of material flows in the circular economy such as that specified by the UN GEO5 report, which states that “In a Circular Economy (CE), material flows are either made up of biological nutrients designed to re-enter the biosphere or materials designed to circulate within the economy via the processes of reuse and recycling” [11]. However, Haas et al. [1] critique the use of such criteria in assessing the circularity of an economy, particularly regarding the notion that all biomass exists in the form of a “circular” material flow. This is because it implies that the production of biomass in any economy is conducted in a renewable manner and that subsequently, all associated waste material flows and emissions can fully reintegrate themselves back into ecological cycles [1]. In reality, when net carbon emissions are considered, factors such as soil nutrient loss and non-renewable water source depletion will render the biomass flow as noncircular, with the exact share of flow that meets the established circularity criteria being difficult to determine [1].

Haas et al. [1] emphasize the notion that additional strategies other than conventional recycling must be employed to achieve circularity in economy-wide material flows, noting that although for materials such as conventional metals and glass, recycling is advanced, in areas such as the construction and demolition industry, considerable efforts are currently in progress to augment recycling rates [12,13]. Additionally, Haas et al. [1] warn that such recycling-based approaches do not lead to an effective reduction of material use since they may have high energy requirements or result in low-quality secondary materials, the use of which will result in an increased demand for virgin material. For this reason, Haas et al. [1] stress the importance of first establishing frameworks on how to assess specific measures and improvements in conjunction with overall contributions to ensure circularity of material flow loops and maximize utilization of ecological material cycles. Essentially, the notion of looping materials around, via traditional recycling pathways, does not necessarily result in an increase in circularity.

An alternative means of approaching this issue is the use of cyclical use rate indicators that express the ratio that secondary materials are consumed in addition to primary raw materials, thus providing an integrated approach for these issues [14]. The basic cyclical use indicator was initiated by the Japanese government in 2003 and was adapted in 2014 by Kovanda et al. for the Czech Republic to take into account the consumption of all secondary recycled materials, as shown in Equation (1) [14]:

(1) $$PU_{cm1+2}=\frac{U_{cm1+2}}{DMI_{-im}+U_{cm1+2}}$$

In Equation (1):$\;PU_{cm1+2}$ is the cyclical use rate indicator as a percentage with modifications made for waste imports, secondary materials, and scrap along with domestically produced secondary materials. $U_{cm1+2}$ is the cyclical use rate of all materials, and $DMI_{-im}$ is the direct material input, excluding waste imports.

Although the aforementioned indicator was used to create a successful EW-MFA Sankey diagram for the Czech Republic context, once again methodological issues were encountered with regard to the selection of which waste treatment methods should be used for inclusion as cyclical use materials ($U_{cm1+2})$ as well as the imports of waste, secondary materials, and scrap whose laborious data collection was reduced and suggested for reassessment by Kovanda et al. [14] in future iterations.

#### 1.2.3. Procurement of Algorithms and Referential Data

An additional challenge to the rendering of dynamic material flows such as that of a circular economy is that of the algorithms and referential data used to calculate material flows between various economic activities [9]. Commonly, material flow modelling depends on input─output analysis (IOA) that assists in linking material flows to their respective economic activities. The data utilized for IOA are extracted from input─output tables (IOTs), which detail interindustry trade of goods and services [9]. When these outputs are integrated into a framework, they enable the modelling of material flows within an economic system. However, experts can only utilize these frameworks, which are not intuitive enough to be easily adopted or used by most of the sustainable material management (SMM) practitioners present in government agencies and their related industries. 

#### 1.2.4. Communication-Based Challenges

During the data gathering process for circular material flows, circular product life cycle (CPLC) stakeholders tend to withhold available product data from other stakeholders present at the end-of-life phase [15]. This is done despite the insistence of production life cycle information sharing among product life stakeholders by remanufacturers [15,16]. Greater efficiency in CPLC information flow is expected to provide various benefits for all product life cycle stakeholders, with the primary benefit being towards customer satisfaction with regard to improvements in product performance and service [15]. To identify specific constraints for efficient product life cycle information flow, a recent study by Kurilova-Palisaitiene et al. [15,16] determined the following constraints between CPLC stakeholders:

- A lack of awareness of a need for circular information flow;
- Underdevelopment of a shared value system;
- Uncertainty and inflexibility in available information;
- Lack of available information due to fears of competition;
- Limited information access on remanufacturing;
- Lack of motives for information sharing with remanufacturers.

**Figure 2:**
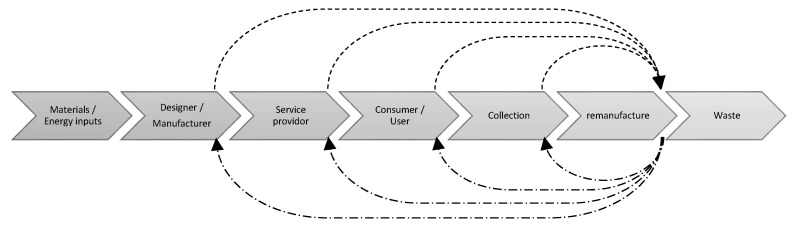
Measuring circularity requires information flow in the feed-forward (dashed lines) and feedback (long dash dot lines) directions across the multitude of circular product life cycle stakeholders. This diagram is illustrative only and multiple other data flows not presented exist amongst the stakeholders.

Additionally, the development of information flow data into a Sankey diagram presentation demonstrated that two main types of information waste hinder the effective CPLC data flow via remanufacturing [15,16]. These are feed-forward information losses and the feedback information bottleneck (Figure 2). Feed-forward information losses occur during the transfer of information towards the remanufacturing sector, with the feedback information bottleneck relating to poorly utilized information regarding remanufacturing feedback to close the loop. This in turn implies that most information created by remanufacturers in the circular product life cycle is not used by other CPLC stakeholders. To address this, Chen et al. [9] and Kurilova-Palisaitiene et al. [15] suggest the adoption of standardized information exchange networks/channels that would assist in facilitating rapid feedback and data exchange opportunities, which would culminate in the creation of a system of shared values via the establishment of a platform that coordinates data and information sharing and ownership.

#### 1.2.5. Interagency Collaboration

The procurement of relevant material life cycle data usually falls within the governance capacities of a variety of government organizations dictating policy in various sectors including agriculture, management, international and national import/export, consumer goods manufacture, and environmental regulations [9,17]. Moreover, barriers may exist that prevent the effective engagement of material flow information amongst different government agencies when their scope of understanding is usually limited to their respective sector [9]. According to Chen et al. [9], one example where a lack of understanding may occur is the procurement of material flow data from design and manufacturing industries, which often possess a limited understanding of the impacts regarding their choice of materials at the end-of-life waste stream. Chen et al. [9] propose that a potential means of mitigating these interagency collaboration issues is to modify industrial standards to engage environmental protection and manufacturing-related agencies. 

### 1.3. Current Circular Economy Policy

#### 1.3.1. New South Wales (NSW)

Government intervention plays a crucial role in developing the circular economy by encouraging economic actors to take a life cycle perspective [18]. More specifically, the use of innovative policy tools that are both fiscal and non-fiscal in nature, such as environmental taxes and levies, subsidies/incentives, permits and regulations, awareness campaigns, etc., help to catalyse circularity in the economy by encouraging businesses to design out waste in the entire material value chain as opposed to conventional end-of-life solutions [18]. Additionally, relevant government policy may also assist in providing financial backing for businesses to generate innovation in the field of circular economy technologies and business practices and leads to greater consumer awareness about material circularity and the emergence of small-to-medium-scale circular economy markets [18]. 

Being the largest economy in Australia, New South Wales is experiencing a range of environmental issues related to above-average economic and population growth and its associated infrastructure development [18]. These include dependence on coal for electricity generation, water shortages, and increases in waste generated [18]. To address these issues, NSW has implemented various upstream and downstream fiscal (e.g., tradeable permits) and non-fiscal (e.g., green public procurement, voluntary agreements) tools [18]. 

New South Wales, having only recently introduced a circular economy policy (in 2018), has created an opportunity for potential policy improvements/augmentations and leapfrogging ahead of international circular economy policies.

#### 1.3.2. Scottish Environmental Key Performance Indicators

The organization Resource Efficient Scotland has created specific environmental key performance indicators (KPIs) that help organizations provide a practical framework to monitor and measure resource usage. These KPIs pertain to various business types (food, hotels, offices, etc.) and relate to energy (e.g., kWh/unit produced in manufacturing businesses), water (e.g., cubic meter per number of staff working in offices), and waste (e.g., tonnes of general waste per occupied room/guest in hotels) [18]. 

#### 1.3.3. German Electrical and Electronic Equipment Act (ELEKTROG)

Regarding downstream non-fiscal tools, the German government has implemented the electronic and electrical equipment act (ElektroG) that aims to ensure economic circularity by holding manufacturers, importers, exporters, and distributors accountable for the entire life cycle of their products [18]. It also holds local governments accountable for consumer waste by making it obligatory to set up municipal electronic waste collection points [18]. Furthermore, the generation of new electronic waste is reduced by requiring retail stores to retrieve a used device of the same type free-of-charge from consumers, upon selling a new electronic piece [18]. 

#### 1.3.4. German REtech Partnership

Currently, in the state of New South Wales, no effective partnership scheme exists to ensure adequate information flow through circular economy stakeholders and, as such, is generally subject to the complications of feed-forward and feedback information losses previously described by Kurilova-Palisaitiene et al. [15,16]. To address these issues, Germany has initiated the REtech partnership (recycling technologies and waste management partnership) that involves active collaboration between government institutions and companies to address waste management issues, such as the improvement of export requirements for companies in the recycling and disposal sector, promoting innovative recycling and efficiency technology, and the sharing of waste management knowledge and expertise. Ultimately, it aims to develop an effective holistic communication network that consists of agencies, scientific organizations, and associations to assist in the export of German recycling and waste management technology in addition to knowledge transfer [18]. 

### 1.4. Recent Developments in Circularity Indicators

Expounding upon the notion of Haas et al. [1] on evaluating the environmental benefits of primarily recycling-based circular economies and their potential detriment, Jacobi et al. [3] suggested “socio-economic cycling rates—the share of secondary materials in total primary materials (ISCr) and in interim outputs (OSCr)—as more adequate indicators to describe circularity”. They believe that adopting such a framework will assist in shortcomings regarding the relevance of biomass and fossil fuels for biophysical economies and augment the understanding of circularity more comprehensively [3]. However, the adoption of such a system is incumbent upon policy and statistical agencies providing detailed, harmonized information regarding resource use and wastage in conjunction with additional research to determine the trade-offs regarding materials, energy, and emissions and their anticipated recycling-based benefits [3]. 

Similarly, Reickhof et al. [19] determined that material flow cost analysis improves integrated assessments methodologically. It was recommended that formalized management control systems and performance measurement systems account for the hidden burdens of nonproduct output, which can then be used to generate high-value products with reduced waste [19]. Further suggestions by Rieckhof et al. [19] included:

- Further development of environmental impact indicators to better define the temporal and geographical occurrences of impacts.
- Updating standards and guidelines, so that product and nonproduct outputs are defined by physical instead of financial criteria.
- Advancing flow-based classification in modelling software to address issues regarding how to best visualize flows in Sankey diagrams.
- Conducting further research to determine when circular economy strategies are desirable and when they are not, in addition to how to mitigate negative effects, as conventional life cycle assessment and material flow cost analysis tend to show a stagnant environmental–economic relationship.

Recently, a general system definition of processes and material flows associated with circular economy strategies within a product life cycle or organization was proposed [20]. It aims to facilitate the monitoring of the circular economy by the creation of an accounting framework that enables the tracing of stock and material flows and enabling their quantification in both physical and monetary terms [20]. It follows a three-layered Sankey diagram format categorized as one of (a–c) [20]:

(a) Comprises material flows/cycles in conjunction with transformation processes that directly relate to the circular economy.
(b) Relates to flows with the economic background system.
(c) Flows with the global socioecological system.

This system definition was based on Graedel et al. [13] and was modified to include biological nutrient recycling and the major circular economy flows developed by the standard BS 8001:2017 [13,20]. The system accounts for upstream natural resources, downstream waste management, direct circular economy flows, and flow losses.

Through the development of the system definition, Pauliuk deduced that a closer link between current circular economy standards, such as BS 8001:2017, and environmental and social impacts, is needed in material flow assessment and accounting. 

Furthermore, the system definition suggested that of the plethora of circular economy indicators currently being researched, the indicators of in-use stock growth, natural resource depletion, and the useful service lifetime of materials should account for core circular economy indicators in future applications and standards [20]. Pauliuk [20] attributed that current incoherency in circular economy standards to a neglect of monitoring the circular economy from a systems perspective, which may eventually culminate in the corporate “cherry-picking” of results to align with a corporate message rather than the long-term goals of the circular economy and sustainability practices. Finally, Pauliuk [20] reiterated the same notion as Brears and Springerlink regarding the inability of organizations to fully implement the circular economy and the need for policy interventions for the support of material efficiency, in conjunction with sustainable development goals and climate targets.

To assist in the separation of economic objectives from ecological objectives, a fundamental objectives hierarchy was recently proposed to determine the relative importance and separation of economic objectives from ecological and social objectives [21]. Velte et al. [21] suggest that the analysis of circular economy objectives through the fundamental objectives hierarchy aims to uncover and sort the objectives to better define the circular economy through its values, and value reasoned objectives that in turn will assist in rectifying gaps between the systemic circular economy objectives and corporate objectives during the decision-making process. However, this hierarchy is limited to a generic understanding of circular economy values and evaluates individual goals before objective personalization in subsequent steps. 

A “closed-loop” circular economy research model was proposed on a more simplified scale that aimed to deviate from the current emphasis on theoretical/technological solutions to new circular business models that engage internal and external stakeholders [22]. The crux of the model is to provide a means for the continuous real-world testing of theoretical circular economy tools whilst using findings from testing to generate new research ideas. This is achieved by the circularity of theoretical goals of the circular economy linking to novel methods and models, followed by strategies for policy and business, then applications across the industry, assessment to inform continuous innovation, and linking back again to informing the aforementioned theoretical goals. 

#### The Longevity Factor

Recently, the measure of longevity was suggested to determine contributions to the circular economy whereby the greater the length of time a resource is utilized, the greater its supposed contribution to the circular economy [23,24]. The approach suggested by Franklin-Johnson et al. [24] states that resource longevity can be determined in three main methods: (A) the time it is initially used, (B) the time used after refurbishment and (C) the time used due to recycling. This led to the development of the formula for longevity shown in Equation (2) [24]:

(2) $$\mathrm{Longevity}=L^{A}+L^{B}+L^{C}$$

where $L^{A}$ is the initial product lifetime, $L^{B}$ refers to the lifetime contribution of refurbished products and $L^{C}$ refers to the recycled lifetime contribution.

Figge et al. [23] note that the above model does not account for varying frequencies of return, refurbishment, and recycling, thus limiting the adaptability of this formula. To address these shortcomings, upon modification of the longevity formula to assume constancy in return, reuse, and recycling rates, and the assumption that all returned goods are recycled, Figge et al. [23] created a combination matrix for longevity and circularity that accounts for the strengths and weaknesses of both approaches. The matrix demonstrates four possible means of combining longevity and circularity and aims to provide strategies for sustainable resource use [23]. Here, the matrix indicates that materials with a high circularity and longevity that fall within the “long circular” segment will significantly contribute to the circular economy [23]. 

Figge et al. [23] suggest the use of distinguishing indicators for (a) initial use, (b) refurbishment, and (c) recycling. These are anticipated to illustrate how to use resources in a long circular manner. Additionally, Figge et al. [23] note that the scope of indicators used are limited to specific product systems and cannot currently accommodate other circularity measures such as open-loop recycling. Thus, they suggest future research to account for the incorporation of the recycling of the resources used to create other products and services within firms.

### 1.5. Further Developments in Material Circularity Research

Hakulinen et al. [25] examined the practical feasibility of small-to-medium-sized enterprises (SMEs) circular business model (CBM) in Finland. The MCI methodology was classed as a comprehensive indicator, whereby owing to the “complexity and comprehensiveness” of the indicator, extensive estimations and assumptions are required. This requirement, combined with a general ambiguity in some areas of measurement, generally requires the assistance of consultancies and experts, something which Hakulinen noted was “too high flown and difficult to apply in practice” [25]. 

They attribute this to the relative lack of data management systems and accounting professionals, which in turn requires indicators to be as simple as possible and standardized for simplification in external reporting, communication, and comparability [25]. Furthermore, they noted the lack of development in the measurement process, suggesting that the automation and integration of circularity indicators into enterprise resource planning and strategic performance measurement systems in companies need to be improved, along with indicator comparability, which limits comparison between industries owing to company-specific modification.

Recently, a simple bidimensional approach incorporating both the MCI and LCA aimed to assess the trade-offs between material circularity and LCA for end-of-life vehicle tire management. Circular economy strategies noted the importance of circular economy tools to focus on micro and macroscale assessment [26]. Assuming a static economic state, the approach aimed at pathway identification from a baseline, regarding the following four areas [26]:

- Coupling reinforcement indicates a stronger dependence on environmental inputs (low material circularity), resulting in more significant environmental impacts.
- Decoupling: this implies an eco-efficient CE strategy.
- Resource trade-off: this suggests the progress made for environmental impacts requires additional resources.
- The trade-off on reservoirs: when saving natural resources costs more environmental externalities.

Furthermore, it was noted that despite their alleged claim to sustainable development, nearly every circularity measure neglects social aspects and economic factors, particularly considering the detrimental effects of Jevon’s paradox that relates increases in product demand with technological improvements, as well as assessing CE improvements via the analysis of both natural resources and pollution reservoir preservation. Finally, additional shortcomings of the MCI were noted, regarding its inability to recognize burden-shifting by the exclusion of energy and background flow, while the use of absolute values and economic factors in future research was suggested [26]. Expounding upon social aspects, Rahla et al. [27] note that despite the social aspect of indicators being commonly overlooked, their high subjectivity results in the overall methodology being weakened [27]. 

Recent studies have explored the modification and combination of existing indicators to fill in gaps in the indicator methodology or transfer to different levels (e.g., micro to meso level). Razza et al. [28] developed a modified MCI to assess bioplastics circularity and found that renewable feedstock was the driver of bio-based and biodegradable products. Although the paper investigated bioproducts degradation, consideration of how these products could be returned to the biosphere to ensure a restorative process could have been made. Further investigations of the relationship between bio-based and biodegradable products and renewable feedstocks would be needed to identify circularity beyond just materiality. 

Lonca et al. [29] combined MFA, MCI, and LCA to assess plastic (PET) bottles’ material efficiency and discovered that closed-loop systems increased material circularity on a product level, but not on a PET market level unless the reclamation rate was increased. The paper did not explore reclamation rates via meso or macrolevel strategies. As product-level CI outcomes can vary from on a meso or macro level, it highlights the need to fill in gaps in understanding CE and indicators’ socio-economic dimensions. This is further highlighted in a study by Harris et al. [30], where environmental impacts using current assessment methods aimed towards a CE were found to be difficult to transfer across other levels. 

Rossi et al. [31] developed a qualitative set of indicators for application to circular business models. Although this indicator set encapsulates the CE’s environmental, economic, and social dimensions, companies may use this qualitative approach to present their data in the best light and may not be truly reflective of CE progress. 

Moreover, the aforementioned lack of consensus on what constitutes a circularity indicator and its subsequent subjective framework to assess circular economy strategies has led Niero et al. [32] to couple different types of indicators via a multicriteria decision analysis method (MCDA) as a means of dealing with metric-based bias [32]. 

By comparing four alternatives for beer in the United Kingdom and Indian markets against the MCI and material reutilization score (MRS) material reuse circular indicators and against the life-cycle-based indicators of abiotic resource depletion, climate change, acidification, particulate matter, and water consumption, Niero et al. [32] deduced that the coupling of indicators via MCDA allowed for the integration of the unique perspectives of the indicators and led them to suggest the use of the technique for order by similarity to ideal solution (TOPSIS) methodology to help better understand comparative methods between complementary indicators [32]. TOPSIS operates via a Euclidean distance measure to identify positive and negative ideal solutions. The former is a hypothetical alternative using the highest score of benefit-type indicators. Likewise, the latter is a hypothetical alternative using the lowest score of harm-type indicators [32]. In the TOPSIS approach, alternatives closest to the positive ideal solution receive the highest score and favourable weighting [32]. 

Assessing the effects of CE strategies is also vital in ensuring sustainable outcomes. Dhanshyam et al. [33] investigated policy mix to mitigate plastic waste in India via a systems dynamics model. Phased kerbside recycling was shown to be the most effective approach when mitigating plastic waste stock, whereas plastic bans were shown to be the least effective. This paper only accounted for eliminating and recycling plastics, whereas other reverse logistics methods could have been considered. Materiality was also the main subject, whereas background processes such as energy consumption also need to be looked at to avoid burden shifting. 

Although circularity indicators aid in policymaking, a forecast of policy implementation will be useful to validate indicator methods. Nano and micro indicators were reviewed by Oliveira et al. [34] for their potential in policymaking. These indicators mainly focused on the material’s reuse stage and “lack robustness to assess the sustainability performance of circular systems”. Thus, current nano and microscale methods have also yet to account for other CE dimensions and realize outcomes on other levels. 

Shi et al. [35] examine the trends and relationships between plastic waste, waste management policies, and international trade networks. Due to global trading’s dependent nature, when waste management policies are changed, other countries are forced to restructure their waste network. Although environmental and long-term impacts could have been assessed, this work reveals how global trading can act as an enabler and disrupter for waste generation. 

### 1.6. Ellen MacArthur Foundation Circularity Indicators Project

In 2015, The Ellen MacArthur Foundation (EMF) launched the Circularity Indicators Project to address the gap in measuring companies’ effectiveness in their journey of transitioning from linear to circular economy models. The project encompasses various indicators, including the main material circularity indicator (MCI), to determine the restorative abilities of product material flows and an additional complementary indicator that provides a platform for further organizational risk assessment for material circularity [36]. According to the Foundation, the uniqueness of the Circularity Indicators Project is that the development of the methodology used involved the active participation of various stakeholders, including European businesses, universities, and investors that collaborated with the project team to develop, test, and refine the circularity measurement system to ensure its practicality and ease of adoption by circular economy stakeholders. 

**Figure 3:**
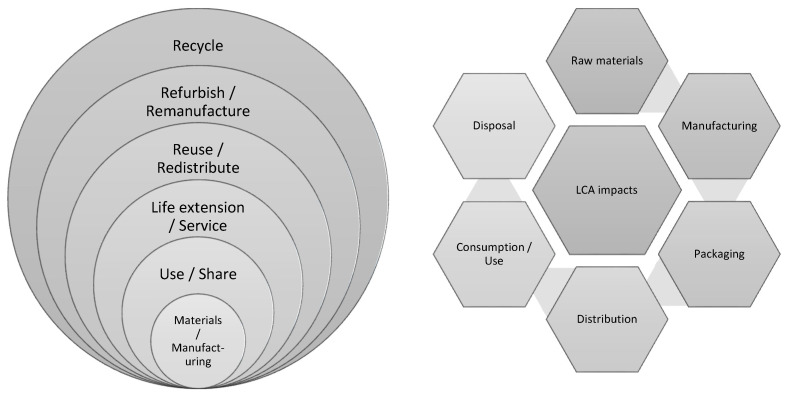
The complementarity between material circularity (**left**) and LCA (**right**). Circularity of a product focuses on the flow of materials during the product lifetime whilst also promoting the recycling and reuse of material via the recognition of product utility. In contrast, LCA aims to primarily derive life cycle environmental impacts of a product via the analysis of multiple scenarios.

Currently, the project only encompasses indicators focusing on technical cycles and materials from non-renewable resources due to their greater ease of understanding [36]. Furthermore, it deviates from conventional life cycle assessment (LCA) methodologies (Figure 3). The MCI focuses on the flow of materials during the product lifetime whilst also promoting the recycling and reuse of material via the recognition of product utility. In contrast, LCA aims to primarily derive life cycle environmental impacts of a product via the analysis of multiple scenarios [36]. With regards to similarities, impact indicators for MCI calculation may be derived utilizing LCA data from the Foundation, suggesting that MCI may be incorporated as a future output for LCA and associated “eco-design” approaches [36]. 

#### 1.6.1. Material Circularity Indicator (MCI)

The MCI approach used by the EMF provides a value between 0 and 1, whereby the latter indicates a higher circularity. This is an arbitrary indicator intended for comparative purposes between product ranges in an organizational product portfolio. Hence its support for complementary indicators. Inputs considered by the MCI include [36]:

- Production process inputs—this encompasses the consumption of virgin, recycled, and reused components as inputs during production processes.
- Longevity and intensity of product use compared to industry average—this accounts for product durability as well as repair, maintenance, and shared consumption.
- Material destination after use—proportion dumped in landfill, reused, or recycled.
- Material recycling efficiency.

To integrate the above data into the MCI calculation, a detailed bill of materials must be provided for all components and materials used. Regarding its primary applications, the indicator can be utilized internally to compare product ranges and departments whilst also allowing for progress tracking on said product ranges and departments for an entire company. Additionally, external parties can use the indicators for comparison between different companies, investment decisions, and the benchmarking of organizations within a specific sector [36]. 

#### 1.6.2. Assumptions

The EMF MCI model was developed with the following assumptions:

- No explicit favouritism of closed-loop systems where recycling needs to return to the original manufacturer.
- Recovered material can be produced to a comparable quality to virgin material-based products.
- No assumed material losses during the preparation of collected products for reuse
- Biological cycles are not considered.
- Product mass is conserved from “cradle to grave”.

#### 1.6.3. Formulae

Hakulinen et al. [25] note that the MCI utilizes the following formulae (Equations (3)–(9)):

(3) MCI=LFI∗F(X)

(4) $$\mathrm{LFI}=\mathrm{Linear}\;\mathrm{Flow}\;\mathrm{Index}=\frac{V+W}{2M+\frac{W_{f\;}-W_{c}\;}{2}}$$

(5) $$V=\mathrm{Virgin}\;\mathrm{Material}\;\mathrm{Mass}=M\left(1-F_{r}-F_{u}\right)$$

F_r_ = Fraction of feedstock from recycled sources
F_u_ = Fraction of feedstock from reused sources
M = Mass of finished product
  (6) $$W_{o}=\mathrm{Mass}\;\mathrm{of}\;\mathrm{waste}\;\mathrm{being}\;\mathrm{landfilled}\;\mathrm{or}\;\mathrm{incinerated}=M\;\left(1-\mathrm{Cr}-\mathrm{Cu}\right)$$
C_r_ = Fraction of mass being collected for recycling at the end-of-use phase
C_u_ = Fraction of mass in component reuse
  (7) $$W_{f}=\mathrm{quantity}\;\mathrm{of}\;\mathrm{waste}\;\mathrm{generated}\;\mathrm{in}\;\mathrm{recycling}\;\mathrm{process}=M\frac{\left(1-E_{f}\right)F_{r}}{E_{f}}$$
  (8) $$E_{f}=\mathrm{Efficiency}\;\mathrm{of}\;\mathrm{recycling}\;\mathrm{process}\;\mathrm{used}\;\mathrm{to}\;\mathrm{generate}\;\mathrm{feedstock};W_{c}=\;M\left(1-E_{c}\right)C_{r}\;$$
C_r_ = Fraction of mass being collected for use at the end of the recycling phase for component reuse
C_u_ = Fraction of mass utilised in component reuse
Ec = Efficiency of the recycling process used for product recycling at the end-of-use phase
  (9) $$F\left(X\right)=\mathrm{Utility}\;\mathrm{factor}=\left(\frac{L}{\mathrm{Lav}}\right)*\left(\frac{U}{\mathrm{Uav}}\right)$$
L = Length of the product use phase
Lav = Industry average of equivalent product use phase
U = Intensity of use
U_av_ = Industry Average Intensity of use

#### 1.6.4. Complementary Risk Assessment Indicators

In addition to the bill of material inputs, the tool used by the Ellen MacArthur Foundation provides an option for consumers to add additional indicators to their MCI model for means of providing further assistance in corporate planning and strategy. Of these additional indicators, complementary risk indicators or complementary impact indicators can be chosen. Complementary risk indicators may assist in forecasting potential threats and opportunities. They can include material price variations and supply chain volatility, whilst complementary impact indicators may help determine the relationship between material circularity and other business practices [36]. Thus, the complementary indicators provide a platform for project prioritization via the risk assessment of materials, parts, and products utilized in the MCI calculation.

#### 1.6.5. Company-Based Material Circularity Indicator

In addition to the conventional MCI calculation tool, the Circularity Indicators Project also contains a MCI calculation tool for company-level circularity analysis [36]. Inclusion of the feature was attributed to the notion that by the improvement of material circularity of company products, the company will possess greater material circularity [36]. To simplify complications arising from documenting entire product inventories, a reference product approach is used by the indicator whereby the MCI is calculated for reference products representing a greater product portfolio. Additionally, the de minimis rule used in the tool disregards products or departments in the company-level MCI calculation whose contribution falls below a user-selected threshold. The weighted average of each reference product MCI then provides a basis for calculating an overall MCI provided that mass or revenue is used as a basis [36]. Similar to the previous MCI tool, complementary factors can be added for project prioritization and risk assessment. 

#### 1.6.6. Potential Improvements to Circularity Indicators

As mentioned earlier, the Circularity Indicators Project does not currently consider renewable sources. Future applications should aim to incorporate biological cycles and renewable sources and incorporate end-of-use materials into other products [36]. Additionally, concessions could be made in future iterations to allow for a more comprehensive approach on downcycling and material quality losses in recycled products, as previously mentioned by Haas et al. [1], and incorporating support for granular levels of recovery such as remanufacturing. The model could also be expanded to determine the material circularity of major projects and cover a broad array of business models such as performance models and secondary market reselling. 

### 1.7. Case Study—Dutch Postconsumer Plastic Packaging

In the Netherlands, there are two main plastic packaging recycling systems, the separate collection of plastic packaging from households through kerbside collection/central drop-off points and the mechanical recovery of PPW from MSW [6,7]. 

Recent research into the Dutch plastic packaging recycling network has been described by Brouwer et al. [6]. The analysis of 173 unique post-consumer plastic packaging samples was subsequently combined to generate an insight into the entire Dutch recycling network [6]. With the use of material flow analysis and material compositional analysis techniques, Brouwer et al. [6] were able to deduce that the total combination of post-consumer plastic packaging for 2014 in the Netherlands amounted to 341 Gg (gigagrams) net from which the entire recycling network produced 75.2 Gg of milled goods, 28.1 Gg of side products, and 16.7 Gg of process waste. From these data, Brouwer et al. [6] were able to deduce that the overall net recycling yield for the plastic recycling network approximated to around 30% [6]. In addition to this, the report determined the end-of-product-life fates of 35 different types of plastic collected within the network regarding their sorting fate as well as the compositional analysis of the milled goods made thereof to assess the composition of polymeric contaminants in the recycling plastic milled goods [6].

Despite providing a holistic analysis of the network, Brouwer et al. [6] noted that in a similar manner to most comprehensive life cycle analyses, the obtained results (comprehensive data can be found within [6]) are only reflective as the model utilized does not account for standard deviation. Additionally, they noted that the net chain recycling yield was limited owing to various factors, including the lack of mechanical recovery of plastic packaging from MSW, low consumer collection response, and poor sorting methods that culminate in polymeric contamination and loss of wrongly sorted packages.

Importantly, Brouwer et al. [6] deduced that the complex nature of the current Dutch plastic recycling network results in true closed-loop recycling being impossible despite attempting to achieve this goal by the washing and milling of recycled plastic products for non-packaging and non-food packaging applications. This leads them to ultimately suggest that instead of utilizing conventional life cycle frameworks such as closed- and open-loop recycling, the analysis of “numbers and facts” plays a bigger role in establishing policy and technological innovation for the circular economy.

Of relevance to current work and recycling policy is the Dutch extended producer responsibility scheme (EPR). In accordance with European directives on packaging/packaging waste, packaging producers must separate and recycle plastic packaging waste [7]. In the Netherlands, “green dot” companies under the *Alfvafonds Verpakkingen* (translated as “Packaging Waste Fund” in English) scheme are responsible for these legal requirements. These operate by collecting feeds from the retail and plastic packaging industry that are dependent on the volume of waste they generate and providing compensation for municipalities who are legally obligated to collect and treat household waste [7]. Here, most companies (except small supermarkets and plastic producers) pay a fixed contribution for products needing plastic packaging, such as household and toiletry items, whilst all collection, separation, sorting, and recycling costs for packaging waste is fully reimbursed by the scheme, with 677 EUR/tonne being compensated for plastic separation in 2015 [7].

It should also be noted that all European EPR schemes involve basic fee modulation to charge differing fees to producers for packaging materials sold with plastic packaging, generally charging significantly higher fees than other packaging types [37]. For the Netherlands, Watkins et al. [37] note that currently, no EPR subsidies exist for different types of plastic packaging such as PET, with the exception that beverage cartons, biodegradable plastic, and deposited bottles from commercial and industrial sources charge lower fees than unspecified general plastic packaging produced for household, commercial, and industrial sources [37]. 

The Dutch plastic recycling network produces washed and milled goods from mechanical recycling methods with plastic that either originates from a separate collection of plastic packaging or the mechanical sorting of products derived from MSW. From here, they are processed into washed and milled goods of the following product ranges [38]:

- Polyethylene terephthalate (PET): derived from items such as soft drink/water bottles.
- Polyethylene (PE): derived from items such as shampoo, juice, and milk bottles.
- Polypropylene (PP): derived from items such as meal trays, laundry, and dishwashing detergent bottles.
- Film: derived from materials such as grocery bags cling wrap, etc.
- Mixed: these are derived from hard plastic sources such as polyvinyl chloride, polystyrene and non-beverage bottle PET, and various residual plastic types left over from the sorting process or plastic products that are subject to compositional restrictions [38,39].

**Figure 4:**
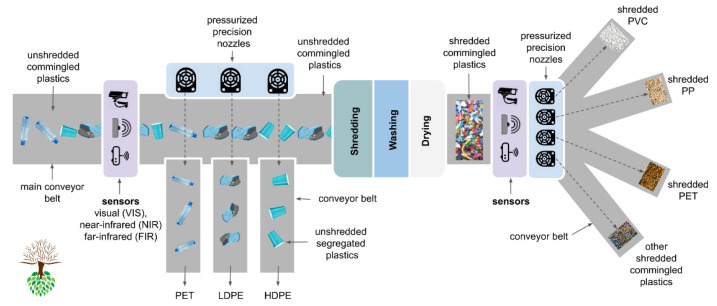
Mixed plastic sorting and processing facility (reproduced with permission from MDPI [41]).

Figure 4 shows an illustration of a generic plastic waste sorting and processing facility. Based on the work of Brouwer et al. [6], the flow of only plastic packages through the PPW recycling network in the Netherlands in 2014 is summarized in Table 1, for the separate collection and collection with MSW.

An example of a PPW processing facilities’ sorting operation is the Suez Automated PPW sorting facility in Rotterdam [40]. From the above-mentioned ranges, the main milled goods are composed of PET bottles of the sinking fraction and the other polymer components’ floating fractions. For the scope of their report, Brouwer et al. [6] disregarded complex additional mechanical recovery processes such as flake sorting and fine sieving. The compositional data of the categories of goods used to contribute to these products are tabulated in their work as: (a) end-of-life fates of 12 different types of plastic packaging waste in the Dutch plastic recycling network, and (b) recovered masses of washed and milled goods predicted by material flow analysis in comparison to their measured values.

### 1.8. Gaps in Research

Although comparative analysis techniques have compared the EMF MCI to other proposed indicators on the macro level [32], the absence of standardized single index methods in current academic research results in an overt emphasis being placed in the current literature on two requirements of the circular economy [4]. Currently, only the MCI aims to account for the loss of materials and product durability/longevity and aligns with Pauliuk’s suggestion for the useful service lifetime of materials accounting for core circular economy indicators in future applications and standards [4]. Despite this notion, apart from comparative indicator examples, the EMF MCI has not been normalized under economic factors for the analysis of product ranges or material circularity amongst any organizations, sectors or projects despite the EMF and Lonca et al. [26] promoting the suitability of the indicator for such applications and as a focus point for future research [36]. Additionally, no aspects of the Dutch recycling network have been subject to assessment by any material circularity indicators similar to the MCI, whose simple index can help identify areas of additional emission reduction, risk assessment, and justification of Dutch policy direction [36].

Moreover, despite the suggestion of Hakulinen et al. [25] that the EMF MCI is a fairly complex circularity indicator, the EMF has simplified the process of company/organizational level integration of the MCI tool. This simplification enables the MCI of a company to be calculated for its products via a reference product approach, whereby only a range of reference products are to be selected for analysis that are representative of the entire product portfolio with smaller product streams being disregarded via the de minimis rule [36]. As a joint venture with Granta Design, the EMF developed a spreadsheet tool that allowed the MCI of different product ranges to be weighed against mass or revenue normalization factors, from which an average MCI is automatically deduced and plotted. This tool operates via the sum product of the normalizing factor multiplied by the MCI divided by the total sum of the normalizing factor. The MCI is automatically plotted against the normalizing factor.

To the best of our knowledge, no currently published work has utilized the “company aggregator tool” to normalize economic factors against real-life MCI data extracted from a life cycle analysis of a product range or assess its effectiveness economic prioritization of product ranges relative to their MCI. The scope of the present work will involve calculating and comparing MCIs for different product ranges, normalizing them against relative economic factors, and discussing the disconnect between economic factors, material circularity, and implementation of the MCI in the business and political realm. 

The objectives of this paper are as follows:

- To calculate the reference MCI for the respective product ranges of washed and milled goods produced from Dutch post-consumer plastic packaging waste.
- To normalize the MCI data against economic factors for the respective product ranges. From here, the MCI for each respective product range can be deduced via the EMF “Reference Product Approach”.
- To assess the practical suitability of the EMF “Company Aggregator Tool” for MCI.
- To discuss the difference between normalized MCI product suggestions and those inferred by conventional life cycle costing/MFA techniques.
- To discuss the policy and business-related implications and potential for the economic normalization of the MCI and circular economy integration.

## 2. Methodology

### 2.1. Material Circularity Index (MCI) Calculation

For the calculation of the MCI, in conformity to the MCI formulae, the following assumptions were made for the MCI calculation:

- For the mass of the finished product (M), the masses of both the main product and side product were added together with the washed and milled product mass data from published datasets by Brouwer et al. [6] for each plastic product type.
- For market reintegration, a product mass comprising of 1% virgin feedstock is assumed (V) due to the lower quality of secondary recycled plastic material, limiting its application [7].
- To determine the fraction of feedstock from recycled sources, (F_r_), the tabulated end-of-life fates (Brouwer et al. [6]) was utilized, whereby the fraction recycled for each product type was determined by the average percentage not recycled based on their ideal sorting fate. This was also assumed to assess the recycling process’s efficiency to produce recycled products (E_F_) and C_r_ regarding the fraction of the product collected for recycling after its end-use phase. These values were assumed to be the same for both MSW and separately collected washed and milled product ranges.
- The fraction of product mass in component reuse (C_u_) was assumed to be 1% in a similar manner to the assumptions made by Niero et al. and EMF in instances where C_u_ is unknown [32,36].
- For the determination of the efficiency of the processes utilized for recycling the product (E_c_), the average values of the measured process waste in the tabulated recovered masses (Brouwer et al. [6]) for each product type were utilized.
- The final washed and milled goods were assumed to have the same average lifetime (L) and number of functional units (U) as those of the industry average (U_av_/L_av_), as suggested by the EMF in instances where the utility cannot be deduced [36].
- The quantities of unrecoverable waste in product production were directly determined by the values given by data from published datasets by Brouwer et al. [6] regarding process waste (W), with all unrecovered waste assumed to be either landfilled or incinerated (W_o_).
- W_c_ or the waste produced when making recycled product parts was assumed to be zero as no parts are explicitly made nor detailed by Brouwer et al. [6]; rather, waste sorted feedstock is washed and milled.
- W_f_ or the unrecoverable waste produced during the production of recycled feedstock was assumed to be the average values of process waste generated during the mechanical sorting process derived from the product and waste mass data from published datasets by Brouwer et al. [6].

### 2.2. Economic Factor Normalization

As no costing information is available in the public domain regarding collection, separation, sorting, and recycling of plastic in the Netherlands, data from Gradus et al. [7] were utilized instead. These data are based on remuneration fees received by municipalities under the *Alfvafonds Verpakkingen* scheme as a “proxy for actual costs” [7]. Under the assumption that private costs for plastic collection and treatment are taken into account, plastic waste recycling collection and transport costs are assumed to be 408 EUR/t with no data being available for a detailed split analysis of the PET, PP, PE, mix, and film washed and milled products as the *Alfvafonds Verpakkingen* program is based on a comprehensive fee that encompasses all individual cost components and product types [7]. Gradus et al. [7] determined that net treatment costs were calculated by the subtraction of revenues from the sale of plastic products by the waste treatment cost, which they deduced to be 269 EUR/t of plastic and comprises of post-collection costs of 204 EUR/t of plastic and revenues of sale and transport of plastics of 65 EUR/t [7]. This results in the subtotal plastic recycling costs to equal 677 EUR/t. The calculated MCIs were normalized relative to their recycling costs and product mass.

## 3. Results and Discussion

### 3.1. MCI Calculation

**Figure 5:**
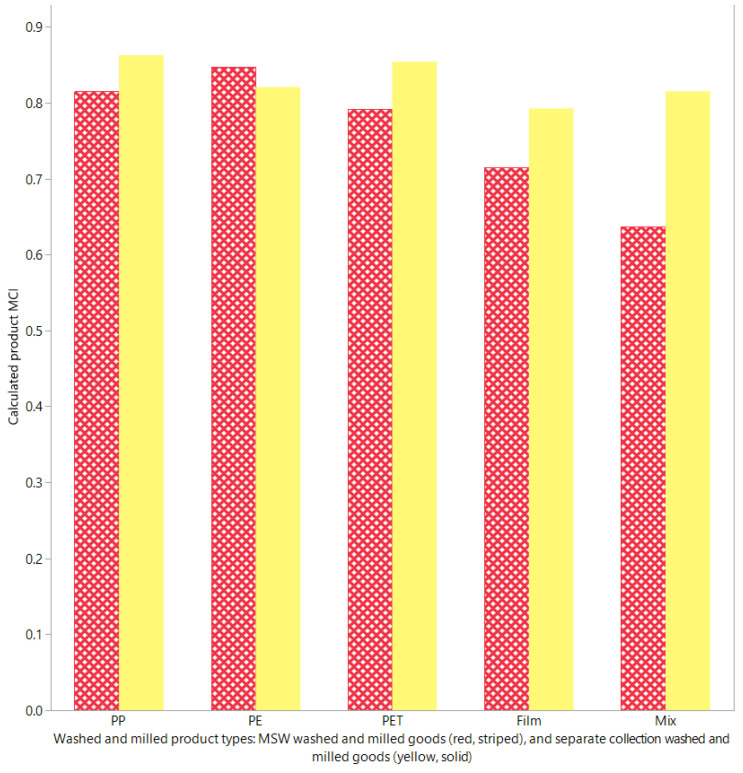
Comparison of calculated MCI for PET, PE, PP, film, and mix washed and milled products produced from MSW and separate waste collection.

Results from the MCI calculations for various product types are presented in Table 2. A graphical comparison of the calculated MCI for PET, PE, PP, film, and mix washed and milled products produced from MSW and separate waste collection is presented in Figure 5. For MSW washed and milled goods, the highest calculated MCI was for PE goods with a MCI of 0.84, followed by PP, PET, film and mixed goods with MCIs of 0.8, 0.78, 0.71, and 0.62, respectively. On the other hand, for separately collected washed and milled goods, the highest calculated MCI was for PP washed and milled goods with a calculated MCI of 0.86, followed by PET, PE, mix, and film goods with MCIs of 0.82, 0.81, 0.8, and 0.78, respectively.

From a direct comparison of the tabulated and graphical results, it is noted that for every washed and milled product type except PE, the calculated MCI of the separately collected products exceed that derived from MSW products. Reasons for this observation can be accounted for as follows:

- PET: the MCI of MSW PET goods was deduced to be 0.78 whilst that for separately collected goods was 0.82. This can be primarily attributed to the fact that a lower quantity of washed and milled goods is derived from the collected packaging relative to the amount of unrecoverable waste produced during the mechanical sorting process (W_f_) with 7.06 Gg of waste produced during the mechanical sorting process to produce 1.9 Gg of washed and milled product compared to 3.3 Gg of waste produced when creating 7.5 Gg of product.
- PP: similarly, the MCI of MSW PP washed and milled goods was deduced to be 0.8 whilst that for separately collected washed and milled goods was determined to be higher at 0.86. This discrepancy can be primarily attributed to the significantly higher portion of average process waste produced during the production process seen in the tabulated recovered masses by Brouwer et al. [6], with an average process waste for MSW measured at 14% in comparison to 4% average process waste for separately collected washed and milled goods. The result is a higher LFI that reduces the MCI.
- Film: the MCI of MSW washed and milled goods was calculated to be 0.71, whilst that of separately collected washed and milled goods was slightly higher at 0.78. Once again, this slight difference can be attributed to LFI increases resulting from a higher portion of process waste produced during the production process at 13% for MSW goods and 6% average process waste for separately collected goods.
- Mix: the lower MCI of 0.62 for MSW goods compared to 0.8 for separately collected goods can be accounted for by the higher quantities of unrecoverable waste (W_o_) produced during both the mechanical sorting and the recycling process of both 5.34 Gg and a W_f_ of 7.06 Gg, respectively, to produce 5.4 Gg of product, thus resulting in a relatively high LFI of 0.376. In comparison, for the production of 35.6 Gg of main and side products, separately collected washed and milled mix goods have a W and W_f_ of 13.8 and 3.8 Gg of waste, respectively, resulting in an LFI of 0.193.

Finally, for PE washed and milled goods, the marginally higher MCI of 0.84 for MSW goods in comparison to 0.81 for separately collected goods can be attributed to the lower LFI of MSW washed and milled goods, whereby a M of 3 Gg of MSW product produces a W_f_ and W of 7.06 and 1.64 Gg of waste, respectively, resulting in an LFI of 0.17, whereas separately collected goods produce an LFI of 0.21 arising from both a W and W_f_ of 3.8 Gg of waste for 8.4 Gg of product.

These results suggest that for the Dutch plastic recycling network, PP PSC possesses the greatest material circularity whilst film washed and milled goods provide the lowest material circularity. On the other hand, for PMSW, it is suggested that PE PSC provides the greatest material circularity whilst mixed washed and milled goods provide the lowest material circularity. For the film and mixed goods for both product streams, their lower MCI calculation aligns with the MFA of Brouwer et al. [6], which notes that mixed rigid plastics such as PVC rigid packaging, laminated flexible packages, PP rigid beverage bottles, and PE miscellaneous rigid packages are subject to lower than average recycling rates—a trend which they attribute to their relatively small object size, which results in them being subject to screening losses during the mechanical sorting process. Furthermore, undesirable and undetectable plastics in conjunction with residual waste present in the stream further limit the amount of washed and milled goods produced. This is further expounded when dealing with the higher rates of undesirable plastic and residual waste present in MSW streams.

For film-based flexible packaging, Jansen et al. [39] attribute their lower recycling rates (and by extension, MCI) to the “insufficiently discriminating nature” of the wind-sifting-based mechanical sorting technologies that are used to separate the flexible film packaging [6,39]. Furthermore, during the separation process, films have the tendency to cover other types of PPW, which in turn results in delays in the sorting process and a reduction in the overall sorting process efficiency [39]. 

Conversely, the higher observed MCIs of PE and PP washed and milled goods also align with the current literature, with Jansen et al. [39] noting that near-infrared (NIR) sorting technologies for PE PPW possess a high sorting efficiency in the sorting of drinking bottle, flask, and rigid PE PPW, with reductions in sorting efficiency (and MCI) arising from the poor sorting efficiency of flexible PE packages that disregard PE packages containing >5% flexible product composition and are detected and removed via manual quality control procedures [39]. Likewise, PP packages are subject to relatively high sorting yields of roughly 85%, with minor sorting inefficiencies arising from the incorrect sorting of composite PP packaging films containing fractions of other polymers and metal contaminants such as aluminium. Furthermore, the incorrect labelling of PET and PE packages with PP labels causes them to be identified and sorted within PP plastic streams, thus increasing residual waste and reducing sorting efficiency [39].

Finally, for PET washed and milled goods, the differences in MCI being lower than both PP separately collected washed and milled goods and MSW-derived PE washed and milled goods also align with observations by Jansen et al. [39], that sorting efficiencies of PET are generally lower than those of PP- and PE-derived plastic packaging waste. Here, it is noted that the yields of impurities such as PP, PS, and PVC are greater than those of comparative PE NIR sorting techniques [39]. Additionally, film contamination in the PET stream in conjunction with poor material preparation and conditioning techniques, combined with NIR mechanical sorting’s technological limitations, also contribute to the lower sorting efficiencies and subsequent MCI reductions. 

### 3.2. Normalization of MCI against Recycling Cost

**Figure 6:**
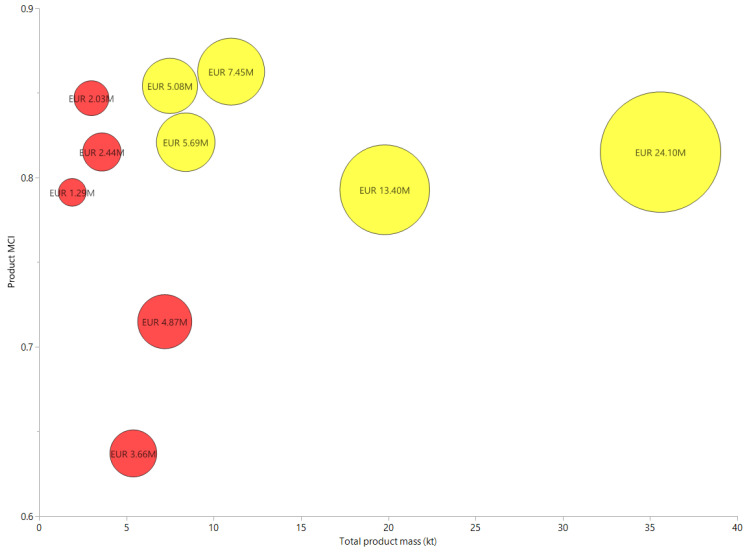
Bubble plot of product MCI, total product mass, and total estimated plastic recycling costs (million euros) for separately collected (yellow) and municipal solid waste (red) Dutch plastic packaging waste (washed and milled goods). Bubble sizes reflect relative scale of total estimated plastic recycling costs (million euros).

A new “circo-economic” indicator, combining product MCI in relation to total product mass, with a cost-normalized total estimated plastic recycling cost (million euros) for both separately collected and MSW is shown as a bubble plot in Figure 6. When the calculated MCI for washed and milled goods are normalized against their estimated recycling cost, there appears to be a clear clustering of data points at lower recycling costs, with higher expenditure in recycling costs providing no noticeable correlations with improvements in material circularity.

For cost prioritization, the option with the lowest recycling cost was the production of PET washed and milled goods, which were determined to have the lowest recycling cost under the costing data assumed by Gradus et al. [7], with a cost of EUR 1,286,300 for the production of 1,900 t of goods with an estimated MCI of 0.78. Conversely, the most expensive recycling option was to produce 35,600 t of mixed washed and milled goods at the cost of EUR 24,101,200, at only a marginal improvement in material circularity over the cheapest recycling option with a MCI of 0.8. Interestingly, the second most expensive option was to produce 19,800 t of film washed and milled goods from the separate collection at the cost of EUR 13,404,600 with the same calculated material circularity of 0.78.

For MCI-based product range prioritization, the previously mentioned highest MCI option of PP separately collected goods at a MCI of 0.86 produced 11,000 t of goods at the cost of EUR 7,447,000, with the next highest MCI option of MSW-collected PE goods providing 3000 t at the cost of EUR 2,031,000.

Similarly, it can be observed that despite comparable levels of material circularity, the amounts of washed and milled products produced from MSW continue to be substantially lower than their separately collected counterparts despite being assumed to cost the same rates to collect, transport, and to process under the all-inclusive fixed fee system of the *Alfvafonds Verpakkingen* scheme.

Indeed, this is the case for every plastic good type shown in Table 2, with 1900 t of PET MSW washed and milled goods being produced at a MCI of 0.78 compared to 7500 t of PET separately collected goods with a MCI of 0.82. 

For PE, 3000 t of MSW washed and milled goods were produced with an MCI of 0.84 compared to a higher quantity production of 8400 t of separately collected goods, despite having a lower calculated MCI of 0.81. This in turn, suggests that the production of PE washed and milled goods from MSW should be prioritized over their separately collected counterparts by Dutch recyclers under the *Alfvafonds Verpakkingen* scheme in future alignment to CE objectives.

For PP washed and milled goods, 3600 t of MSW sourced goods were produced with an MCI of 0.8 compared to a noticeably higher MCI of 0.86 to produce 11,000 t of the separately collected product range. Finally, regarding film and mixed goods, 7200 t of MSW film washed and milled goods were produced with a MCI calculation of 0.71 compared to 19,800 t of separately collected goods with a MCI of 0.78. For mixed goods, a significant disparity in both the MCI and product mass was noted, with 5400 t of MSW goods being produced with a low MCI of 0.62 compared to 35,600 t of separately collected goods with a substantially higher MCI of 0.8.

Upon comparison of the cost/mass-normalized MCI against the average MCI for MSW and separately collected washed and milled goods, a slight reduction in the normalized MCI for cost was observed for MSW, whilst average and cost-normalized MCIs were calculated to be the same for separately collected washed and milled goods.

Overall, for each product range, under cost and mass normalization with the aggregator tool, the overall calculated MCI for both product ranges in both normalization methods was the same, owing to costing rates being held constant with a normalized MCI of 0.73 for the total production of 21,100 t of MSW washed and milled goods at a total cost of EUR 14,284,700 and an MCI of 0.81 for the production of 83,200 t of separately collected washed and milled goods at a total cost of EUR 55,717,100.

### 3.3. Perspectives and Limitations

Under the directive of the EMF “Company Aggregator Tool”, the normalization of the MCI against cost and product mass should act as suitable normalizing factors for the organizational prioritization of washed and milled plastic product ranges. However, owing to the inability to procure accurate costing data regarding the total cost required to process washed and milled goods from PPW, cost normalizations were made equivalent to product mass MCI normalizations, which ultimately suggested that separately collected washed and milled plastic goods possess a higher overall MCI when normalized against their product mass. This would deceptively lead consumers to conclude that conventional recycling schemes that emphasize separate waste collection best align with the CE goals.

As previously mentioned by Lonca et al. [26], the EMF MCI methodology contains a narrow system boundary definition that prevents the effective analysis of the implications associated with macroscale resource transfer owing to its overt emphasis on single foreground level resources, which in this case is post-consumer PPW. This, in conjunction with the MCI’s neglect of background energy, flows such as fuel consumption, and the energy offset from PPW incineration, suggests that the assessment of the above results with additional considerations may provide a better insight into the determination of the recycled product range with the greatest material circularity. This aligns with the suggestions of the EMF, who recommend further investigation into the MCI calculations, should the user believe that the overall MCI is not reflective of a product range place in a product portfolio [36]. 

Furthermore, despite claiming that the EMF MCI does not favour closed loops, the EMF notes that the use of closed loops enables greater purity in material streams, which improves recycling efficiency and by extension the calculation of the MCI [36]. However, Brouwer et al. [6] note that the complexity of the PPW recycling network with its consideration of side products, residual wastes, and nonpackaging plastics ensures that cradle to cradle or closed-loop recycling remains “almost impossible” with the majority of the intended washed and milled goods ending up in “non-packaging and non-food packaging applications” [6]. 

Indeed, in support of this notion, previous research has identified discrepancies in separate plastic collection, with Gradus et al. [7] noting that its collection and transportation costs is “substantially higher than for normal (MSW) waste”. Gradus et al. [7] attribute this to the lower density of separately collected PPW than conventional MSW that facilitates additional transport per tonne than for denser MSW streams. Additionally, as unique infrastructure or kerbside collection points are needed to procure separately collected PPW, substantially higher capital and operating costs arise for the additional expenses associated with the procurement of trucks and PPW collection personnel [7]. This coupled with the typically lower volumes of separately collected PPW result in realistically higher costs per unit of PPW collected [7]. Noting these substantial collection costs, it is estimated that the *Alfvafonds Verpakkingen* scheme allocates approximately two-thirds of all rebates to PPW collection and transport costs [7].

Although accurate data are not available regarding the costing implications of the above factors, conducting a sensitivity analysis under reasonable assumptions can be used to examine the robustness of cost or MCI deviations on the normalized MCI, for separately collected/MSW PPW to validate the credibility of the authors’ hypothesis that the MCI of MSW-derived washed and milled goods is comparable to separately collected PPW washed and milled goods and that they should be incentivized by recycling schemes.

### 3.4. Sensitivity Analysis

The sensitivity of economic factors on the cost-normalized MCI is determined in three scenarios (including an assumed baseline), and results summary are presented in Table 3.

In the baseline scenario, data from Brouwer et al. [6] are assumed to be representative of the quantities of MSW and separately collected washed and milled goods from PPW in 2014, at the cost of 677 EUR/t for the collection and processing of both PMSW and PSC.

For the first sensitivity scenario, Gradus and Dijkgraaf et al. note that for the Netherlands, transport and collection costs could be most effectively reduced by the introduction of unit-based pricing for mixed/compostable waste for the facilitation of increased separate collection of recyclables [7,42]. This, along with separate bag/bin collection, is expected to significantly increase volumes of collected PPW [7,42]. In light of this, Gradus et al. [7] estimate that in 2019, the expected *Alfvafonds* remuneration fee will be reduced to 557 EUR/t owing to reductions in collection costs, with the reduction anticipated to be a lower bound estimate and collection and post collection remuneration assumed to be 299 and 193 EUR/t of plastic waste, respectively. As no English language source could be found to provide any information regarding the 2019 updated fee, Gradus et al. [7] is assumed to hold true in this scenario. Nevertheless, even with the substantial improvements in the reduction of collection and transport costs anticipated in 2019, the cost-normalized MCI for both product ranges will remain unchanged due to the MCI’s neglect of background material and energy flows in the determination of the product range MCI. However, as improvements in collection and transport align with higher volumes of plastic collected and produced, and by extension, higher levels of product manufacturing, if a modest 5% increase in the total product mass for each product range is assumed, the cost-normalized MCI slightly increases to 0.82 for separately collected washed and milled goods, whilst increasing to 0.74 for MSW washed and milled goods.

Regarding the second scenario, PET is recycled in large enough volumes to facilitate the production of rPET products to a level similar to closed-loop recycling with the production of bottles and trays and nonpackaging rPET products in an open-loop manner [6]. Ideally, bottles for rPET should be free from molecular contamination, possess a high polymeric purity and have polymeric chain lengths capable of restoration [6]. Brouwer et al. [43] note that the biggest challenge in the sorting of PET via mechanical recovery is the presence of polymerically contaminated non-food flasks and the need to implement technologies that are capable of automatically identifying and removing these contaminated items at high rates [6,43]. Although currently under research, the incorporation of tracer technologies for the sorting of PET in MSW appears to be promising, with Brouwer et al. [43] suggesting the selection and implementation of effective tracer technologies as soon as possible. Disregarding technical factors such as the speed of recognition and the appropriate stages for marker removal for the prevention of faulty sorting, in this scenario, a Dutch national scheme is introduced to implement tracer-based PET sorting of MSW. Here, it is assumed that the *Alfvafonds Verpakkingen* rates stay the same and fully reimburse the procurement and operating costs by recyclers.

Compositional data were also obtained from Brouwer et al. [6] for municipal solid waste processed at municipal recycling facilities (MRF) and the subsequent products derived thereof. Based on these data which dictate the average amounts of washed and milled rPET product made from MSW-derived PET beverage bottles, a modest increase of 10% additional product is assumed to recover clear and coloured PET beverage bottles by the implementation of tracer-based PET sorting technology. In turn, this will increase the average quantities of PET-sorted product made by a total of 0.1306 Gg, resulting in an increased MCI of 0.79 for PET MSW-collected washed and milled goods whilst resulting in the overall cost/mass-normalized MCI for MSW washed and milled goods remaining unchanged. Once again, this can be attributed to the marginal increase in overall MSW product mass and the MCI bias towards higher product mass.

These observations support the initial results obtained and suggest that under assessment from the EMF MCI, the cost-normalized MCI of PSC exceeds that for conventionally derived PMSW and better aligns with the circular economy’s goals. Furthermore, despite the higher assumed costs of separate plastic collection and transport infrastructure, the higher overall process efficiencies, reduced presence of polymeric contaminants, and sorting waste production combined with the significantly higher product output of separate collection result in the system boundaries and parameters of the EMF MCI favouring separate collection. This product weight bias results in the implementation of any improvements to marginally improve the MCI of MSW-collected washed and milled goods in scenario-based analyses being negated. Despite these shortcomings, the cost-normalized MCI graph results demonstrate that the MCI of PMSW is not substantially lower than separate collection and even exceeds PSC on comparing certain product ranges such as PE. As such, these datasets certainly warrant further investigation to compare the MCI of the product ranges under different parameters and provide evidence for the Dutch recycling scheme to incentivize MSW plastic recycling when noting their reduced transport and infrastructure costs [7]. 

### 3.5. Feasibility of Business Implementation

Despite the suggestion of higher MSW product yields, there is opposition towards weight-based PPW recycling metrics and benchmarked product yields in the Dutch recycling industry [44]. This can be attributed to the perception that increases in recycled product packaging mass compromise product economics due to their subpar quality and lower demand for plastic packaging derived from virgin material [6,44]. Moreover, the weight-biased MCI benchmark prioritization only promotes end-product optimization with the market increase in MSW plastic goods, resulting in the neglect of consumer and supply chain dynamics. Worrell et al. [44] note the importance of considering consumer demand for recycled plastic as the market underperformance of packaging may prove a greater environmental detriment than the disposal of excess plastic packaging [44]. 

#### 3.5.1. Policy Restrictions 

Previous projects by Nedvang, the Dutch central organization for optimizing packaging prevention, noted that despite recyclers expressing interest in reducing PPW, there remained a general industry complacency into research for the optimization of PPW [44]. Should the EMF MCI measure be implemented, it would be subject to the following notable complications:

- Laxed enforcement: currently, in the Netherlands, both recycled and virgin-derived plastic goods are subject to self-declared compliance measures with no additional reporting needed until being explicitly required by enforcement agencies [44].
- Lack of awareness: in the event of MCI assessment legislation being implemented, Worrell et al. [44] suggest that actors in the recycling industry subject to the Dutch packaging decree will have to be self-informed of such legal changes [44].
- Product information nondisclosure: currently the largest factor limiting the reporting of plastic recycling, under the Dutch SVM pact, technical plastic product data such as composition, weight, production and recycling/disposal costs, and even plastic packaging weight reductions are classed as sensitive information or “trade secrets” [44]. This implies that even if the EMF MCI was to be voluntarily adopted by recyclers, the ability of complete control over the data, and assumptions they base their calculations on, may lead to risks of “greenwashing” or the deliberate miscommunication of MCI data for products. Worrell et al. [44] note that the liberal nature of Dutch plastic nondisclosure laws creates issues for even Nedvang data procurement, citing limited Dutch plastic waste case studies and excessively long development periods for plastics research [44].

#### 3.5.2. Potential Future Research

The comparability of suggestions by Niero et al. [32] to utilize their aforementioned MCDA methodology for the assessment of other CE sectors such as waste and electronics, with the ambiguity in CE definitions further justifies the need to assess CE strategies via the consideration of multiple factors [32]. Furthermore, Niero and Elia et al. [4,32] both note that the assessment of CE strategies through a single dimension, which in this instance was the EMF MCI for Dutch PPW, represents an explicit limitation in the evaluation of CE indicators [4,32]. Here, the MCDA methodology could have been utilized to assess the MCI of the MSW and separately collected product ranges, against other normalization factors for the company aggregator tool for the MCI normalization, or against different life cycle assessment indicators such as the product environmental footprint category (PEFCR) indicator. This has the notable advantage over the EMF MCI of considering foreground and background material flows in the assessment of life cycle stages and their associated impact categories, and thus may provide greater insight into the environmental concerns of Gradus et al. [7] regarding the separate collection of PPW [32,45]. 

It is also noted that further benefit could be obtained from the examination of calculated MCIs normalized against social indicators, something that both Lonca and Niero et al. [26,32] prioritize as needed for research in the assessment of CE metrics, but is currently hindered by the ambiguity in the definition and assessment of social impact indicators. Furthermore, this is further complicated by Hakulinen’s findings, which state that the current literature emphasizes aspects that are linked to the CE but not explicitly on material circularity [25]. To help address this, it is suggested that in a similar means to the contribution of Hakulinen in addressing the research gap for the key circular economy performance indicators that small-to-medium-sized businesses may utilize, a weak market test should also be conducted. This can comprise questionnaires, whereby small, medium, and large PPW MSW and separate collection sorting and processing facilities provide feedback regarding the functionality and practical suitability of social impact indicators and the likelihood of their implementation. Not only will this provide an insight into key future circular economy performance indicators for the normalization of the MCI against social impact factors, but may also assist in the identification of irrelevant social impact indicators and further developments that may be made to existing indicators to expedite their adoption in the CE [25].

Noting the importance of social impact indicators for MCI normalization, the EMF has developed a chart highlighting relevant social indicators, which may be suitable as a starting point for developing a future weak market test when used in complement with the MCI [36]. Subcategories of this social category include human rights (investments, etc.), product responsibility (compliance, etc.), labour practices and decent work (employment, etc.), and society (anticorruption, etc.). Additionally, the EMF notes that when it comes to the use of complementary indicators, organizations may utilize indicators that are already established at a company level, with frameworks such as the global reporting initiative (GRI) dictating the reporting of indicator use with businesses, stakeholders, and the general public [36].

## 4. Conclusions

This work utilized Dutch plastic packaging waste data to calculate the EMF MCI for PE, PET, PP, film, and mixed plastic washed and milled goods, derived from separately collected and MSW-derived plastic packaging waste. We determined that for all plastic product types except PE, the MCI of separately collected washed and milled goods exceeded those derived from MSW. 

For the first time, this work formulated a cost normalization of the EMF MCI under recycling costing data. We determined that the overall cost-normalized MCI for separately collected goods exceeded that for MSW with a MCI rating of 0.73 and 0.81, respectively, thus suggesting the greater CE conformity of separately collected washed and milled goods under cost normalization. 

Sensitivity analysis to determine the effects of anticipated collection cost reductions and technological improvements in MSW PET product yields demonstrated no overall changes to the cost-normalized MCI. This was attributed to the EMF MCI inability to consider background energy and material flows associated with different life cycle stages and its bias towards product mass and process waste production. Sensitivity scenarios involving the general reduction of recycling and collection costs and the use of rPET sorting technologies provided marginal improvements in the MCI of MSW PET product ranges. This suggests that improvements in plastic sorting technology and policy incentives that enable the production of MSW washed and milled goods at levels comparable to their separately collected counterparts may significantly improve their MCI. This, in addition to the savings in transport and collection costs associated with MSW, provides credence to the notion that the production of washed and milled plastic goods from MSW may eventually prove to be a cheaper option with greater material circularity than separate waste collection, especially when combined with improvements in PPW polymeric contaminant control [43]. 

Owing to the lack of publicly available costing and plastic specification data, in the Netherlands, efforts should be made on a legislative and business level for greater transparency in the release of data regarding product specifications and recycling and processing costs for recycled plastic packaging. This, combined with greater diligence by agencies in environmental compliance and greater industry awareness methods such as websites and databases, will enable consumers to trace the materials to source and make better-informed choices regarding the types of plastic they consume and the method through which they dispose of it. In line with Jevon’s paradox, this will help to optimize the recycling of plastic packaging waste with the highest material circularity with anticipated increases in plastic demand owing to global population growth. 

To validate the findings of this work, future research could incorporate industry collaboration with willing PPW recycling companies, for the procurement of accurate economic and material processing data to validate these findings for the Netherlands as well as other regions and to identify trends and methods in the collaborative improvement of plastic packaging recycling and plastic product prioritization. Additionally, as social impact indicators remain in their infancy in the current CE research, further normalization of MCI data against suitable social indicators may provide further insight into consumer plastic consumption and plastic packaging waste disposal habits. This may assist in accounting for trends such as the disparity between Dutch rural and urban plastic recycling rates, with methods such as a weak market test for consumers helping to identify suitable social impact indicators for future MCI normalization [25].

## Figures and Tables

**Table 1:** Flow (net weights) of only plastic packages through the PPW recycling network in the Netherlands in 2014, at the gate of the mechanical recycling step for both the separate collection and collection with MSW [6].

Packaging Waste Type	Separate Collection (Gg)	Collection with MSW (Gg)
PET	7.5	2.0
PE	8.4	2.8
PP	9.7	3.0
Film	18.0	7.0
Mixed	31.5	4.9
Rest	10.9	35.3
Total	86.0	55.0

**Table 2:** Calculated material circularity indicator (MCI) and recycling costs for Dutch plastic-packaging-waste-derived washed and milled goods from municipal solid waste (MSW) and separate collection.

MSW Washed and Milled Goods				Separate Collection Washed and Milled Goods			
Product Type	Total Product Mass (T)	Total Estimated Plastic Recycling Cost (EUR) (Gradus et al. [7])	Calculated MCI	Product Type	Total Product Mass (T)	Total Estimated Plastic Recycling Cost (EUR) (Gradus et al. [7])	Calculated MCI
**PET**	1900	1,286,300	0.78	PET	7500	5,077,500	0.82
**PE**	3000	2,031,000	0.84	PE	8400	5,686,800	0.81
**PP**	3600	2,437,200	0.8	PP	11,000	7,447,000	0.86
**Film**	7200	4,874,400	0.71	Film	19,800	13,404,600	0.78
**Mix**	5400	3,655,800	0.62	Mix	35,600	24,101,200	0.8
**Total**	21,100	14,284,700	0.73	Total	83,200	55,717,100	0.81
**Average MCI**		0.75		Average MCI		0.81	
**Cost-normalized average MCI**		0.73		Cost-normalized average MCI		0.81	

**Table 3:** Comparison of the cost-normalised MCI for separately collected, and MSW washed and milled goods under different sensitivity scenarios.

	Cost-Normalized MCI For Separately Collected Washed and Milled Goods	Cost-Normalized MCI for MSW Washed and Milled Goods
Baseline Scenario	0.81	0.73
Scenario 1: Reduction in remuneration associated with reduced transport and collection costs in 2019 + 5% increase in product mass	0.82	0.74
Scenario 2: Implementation of PET tracer technology for the mechanical sorting and separation of PET from MSW	0.82	0.73

## Data Availability

Not applicable.

## Abbreviations

CE	Circular economy
CPLC	Circular product life cycle
EMF	Ellen MacArthur Foundation
MCI	Material circularity indicator
MCIE	Cost-normalized material circularity indicator
MSW	Municipal solid waste
PE	Polyethylene
PET	Polyethylene terephthalate
PMSW	Plastic products derived from municipal solid waste (washed and milled)
PP	Polypropylene
PPW	Plastic packaging waste
PVC	Polyvinyl chloride
PS	Polystyrene
PSC	Plastic products derived from separate collection (washed and milled)
